# COVID-19 Barriers to Care for Pregnant Patients in Prolonged Isolation

**DOI:** 10.1155/2020/8847859

**Published:** 2020-08-25

**Authors:** Mary E. Burgoyne, Emad A. Elsamadicy, Liviu Cojocaru, Andrea Desai

**Affiliations:** Department of Obstetrics, Gynecology & Reproductive Sciences, University of Maryland School of Medicine, 22 S Greene Street, Baltimore, Maryland 21201, USA

## Abstract

Severe acute respiratory syndrome coronavirus 2 (SARS-CoV-2), the agent responsible for coronavirus disease 2019 (COVID-19), continues to have a devastating impact on healthcare systems worldwide, and many questions remain unanswered. The effect of COVID-19 on the pregnant population is widely debated, and the unique risks in pregnancy have not yet been elucidated. What has been established, however, is the recommendation for healthcare workers to use personal protective equipment (PPE) for both contact and airborne precautions to prevent transmission of the pathogen—adding another barrier to care for vulnerable populations. We report a case of a young woman from Haiti during her first pregnancy, who was admitted to the antepartum service at 22 weeks of gestation with preterm premature rupture of membranes (PPROM) and remained admitted in isolation, though asymptomatic, for over six weeks due to persistent positive SARS-CoV-2 testing. Our case highlights the unique barriers to care that COVID-19 poses to antepartum patients, particularly in the setting of pregnant women with persistent positive testing.

## 1. Introduction

While there are several ongoing investigations into the effects of COVID-19 on pregnant women, there are no published reports on the susceptibility to SARS-CoV-2 in this population or the unique management of asymptomatic patients who remain admitted in isolation. It has been established globally that contact and airborne precautions along with PPE are required in hospitals to reduce viral transmission [1]. An unfortunate consequence of such stringent precautions is that many patients are receiving care through audio and video interface platforms, and physicians have limited direct interactions with patients. Strict visitor restrictions are aimed at tempering the spread of what is known to be a contagious virus [2]. This leads sick patients with COVID-19 or even asymptomatic SARS-CoV-2 carriers to be completely isolated during what is likely to be a frightening time in their lives. Fear is exacerbated in pregnant patients who are also worried about their unborn child's health and safety.

The communication barriers that result from lack of direct interactions due to patient isolation can lead to negative experiences with the healthcare system and adverse patient outcomes. Communication challenges can be further complicated by baseline language barriers as well as cultural differences between patients and providers [3]. These obstacles to quality care become more problematic when providers must convey the likelihood of adverse outcomes to patients and explain the treatment options for a diagnosis that may be especially devastating.

In this case report, we will describe a primigravida migrant patient infected with COVID-19 who remained in prolonged isolation. We will examine the challenges that she and her healthcare team faced daily in achieving quality care in the setting of physical isolation, language barriers, cultural differences, and high risk of adverse maternal and fetal outcomes. The aim is to shed light on the detrimental impact of isolation in patients testing positive for SARS-CoV-2 to emphasize the need for further studies to determine the natural history of the disease and identify whether there is a genuine need for prolonged isolation in asymptomatic patients.

## 2. Case Presentation

Our patient is a 36-year-old gravida 1 para 0, previously healthy female who was confirmed to have preterm premature rupture of membranes (PPROM) at 19 weeks of gestation. The patient provided consent for her case to be presented in publication with the understanding and hope that it would be used to further our knowledge of COVID-19 and improve our management of this virus in the pregnant population. She emigrated from Haiti two years ago. Her native language is Haitian-Creole, and she does not speak, read, or understand English. After counseling at an outside hospital about previable PPROM, she elected for expectant management with plans for admission at 22-week and 5-day gestation. Her pregnancy was otherwise only complicated by iron deficiency anemia.

Upon admission to our tertiary care center at 22 weeks and 5 days, her only complaint was of clear leakage of fluid. She denied any uterine contractions, vaginal bleeding, or abnormal vaginal discharge. She experienced one month of intermittent dry cough, but she did not have any associated fever or chills, chest pain, shortness of breath, congestion, gastrointestinal symptoms, anosmia, or ageusia. She had not recently traveled, and she denied any known local exposures to people with COVID-19.

The perinatology and neonatology teams jointly counseled the patient regarding the risks of periviable PPROM—including the risk of associated fetal pulmonary hypoplasia—and explained different options of care. They also informed her about the standard management of PPROM, which entails continued inpatient admission until delivery due to increased risk of fetal and maternal infection requiring prompt delivery. This counseling was performed using a virtual interface on an iPad, with a virtual Haitian-Creole interpreter, and required multiple conversations over the course of several weeks. After a thorough discussion with the patient and her husband, she elected for inpatient admission, latency antibiotic therapy beginning at 22 weeks and 5 days, and antenatal corticosteroids and fetal monitoring starting at 23 weeks of gestation. Her fetal anatomy ultrasound evaluation was normal one week prior to admission.

On physical examination, she was afebrile and normotensive, with a normal respiratory rate and normal oxygen saturations (>95% on room air). Her cardiac exam was unremarkable, and lung auscultation was clear throughout all lung fields. Her cervix was visually assessed and noted to have no dilation with clear normal fluid leakage from the cervical os. On admission, a bedside ultrasound revealed that the estimated fetal growth was appropriate for gestational age (estimated fetal weight 506 g, at the 39^th^ percentile). The presentation was cephalic, and oligohydramnios (maximum vertical pocket 0.59 cm) was noted, as expected with PPROM.

Due to a departmental policy regarding COVID-19 testing for all admitted patients and given her history of cough, a Roche SARS-CoV-2-RNA rapid test was performed on a nasopharyngeal swab that was collected and resulted positive. In response, the patient was transferred to a negative pressure room on our obstetric care unit (OBCU) and placed on telemetry. Reflexive testing was performed, including a chest X-ray (CXR), complete blood count (CBC), lactate dehydrogenase (LDH), ferritin, triglycerides, aspartate aminotransferase (AST), alanine aminotransferase (ALT), and cardiac troponins. Her labs were normal; however, her CXR demonstrated “mild hazy opacities at both lung bases without consolidation.”

The patient was closely monitored for the development of a fever, oxygen desaturations, and increased work of breathing. Her negative pressure room included a video monitor to minimize direct contact. Antepartum management, as outlined above, included latency antibiotics for seven days, antenatal corticosteroids, and daily fetal monitoring with fetal nonstress test (NST). Her dry cough resolved on day 3 of admission. On day 6, the patient had intermittent desaturations to 92-93% overnight during sleep and required 1 liter of supplemental oxygen. She was able to come off supplemental oxygen later that day and was saturating well on room air.

The Roche SARS-CoV-2 RNA rapid test on a nasopharyngeal swab was performed on our patient every seven days following admission, per our hospital's infection control guidelines. By the third week of testing, the patient expressed frustration and sadness that she must go through the discomfort and pain of swabbing when she was asymptomatic. There were concerns that her mood, engagement, and appetite were being affected by prolonged isolation.

During her admission, she had a follow-up detailed antenatal ultrasound that demonstrated compromised fetal lung development (small chest and increased cardiothoracic ratio). The care team organized a video conference with the patient, her husband, a Haitian-Creole interpreter, and the perinatology and neonatology medical teams. During the interdisciplinary meeting, there was an extensive discussion to educate the patient regarding pulmonary hypoplasia and the poor neonatal prognosis involved with this diagnosis. After discussion, the patient expressed that she and her husband had “always wanted a child and will do everything necessary to give the baby a chance.” She felt that “if a woman is pregnant and the fetus is still living, everything should be done to keep the fetus alive.”

At this point, the patient felt that her “doctors were telling [her] not to have hope for [her] baby.” She expressed how difficult it had been to cope with the prognosis of her baby while isolated in a negative pressure room for weeks and communicating only through iPad screens and virtual interpreters. She worried that her wishes and concerns were being lost in translation. Ultimately, the patient decided to continue expectant management of PPROM until 34 weeks of gestation, at which time delivery would be recommended with a Neonatal Intensive Care Unit (NICU) team present to resuscitate as indicated.

Our patient had positive SARS-CoV-2 RNA tests for six consecutive weeks despite the resolution of mild symptoms during her first week of admission. After the first week, the patient did not demonstrate any overt signs of infection and continued to saturate well on room air. After multiple discussions with the infectious disease medical team, the decision was made to remove enhanced contact and droplet precautions after 42 days of hospitalization without symptoms, with no plans for further testing. The patient developed preterm labor at 29 weeks and five days gestation and underwent an uncomplicated vaginal delivery.

Our patient gave birth to a female with Apgar scores of 8 and 8, at one and five minutes, respectively. Birth weight was 1450 g and weight-to-age percentile was 75%. Neonatal testing for SARS-CoV-2 was negative at 24 and 48 hours, making the neonate no longer a person under investigation for COVID-19. The neonate was admitted to the NICU for over 6 weeks, while our patient was discharged on postpartum day one. The neonate's admission has been complicated by septic ileus, feeding intolerance with bilious gastric output, high frequency jet ventilation in the setting of increased work of breathing and pneumothorax requiring bilateral chest tube placement, and continued failure to wean from supplemental oxygen. The patient continues to advocate for her daughter to receive all possible interventions to prolong life. She has been able to visit the neonate in the NICU a few times per week, and otherwise, she remains at home in self-isolation. The neonate has recently been transferred to a hospital closer to her parents' home in the coming days, where she will continue to require intensive care.

## 3. Discussion

This case leaves the healthcare team with one fundamental question: How does a diagnosis of COVID-19 or identification as a SARS-CoV-2 carrier lead to decreased quality of patient care? The patient's case was challenging because she was an immigrant in an unfamiliar hospital system, a non-English speaker requiring interpreter services, she was pregnant with her first child, and her pregnancy was complicated by previable PPROM at 19 weeks with high risk for fetal mortality.

When patients test positive for SARS-CoV-2, the recommendation is to place them in a negative pressure room with strict requirements for contact and airborne precautions as well as PPE to prevent viral transmission [4]. This recommendation helps to protect other hospitalized patients as well as healthcare providers from contracting the virus and spreading it to other patients. In the case of our patient, it is crucial to consider the effect of isolation on patient well-being. Numerous studies have examined the psychological impact of isolation for contact precautions among patients. Being placed on isolation has been associated with shorter and fewer interactions with healthcare workers, more depressive symptoms, prolonged hospital stay, and lower standards of care [4]. A case-control study investigating the effect of isolation on hemodialysis patients with multidrug-resistant organism colonization demonstrated that adopting contact precautions and isolation can further negatively impact social functioning, sleep, and quality of life [5]. Not only was the isolation for our patient personally demanding in the setting of the novel coronavirus and pregnancy, but her experience as a non-English speaking immigrant also amplified her isolation.

The cultural shock of being an immigrant in an unfamiliar healthcare system that uses an unknown language is certainly isolating. The added precautions for COVID-19 can exacerbate this feeling of distance and loneliness [6]. Moreover, there is evidence that immigrant women are more likely than nonmigrant women to have negative experiences of the healthcare system [6]. Language barriers have been shown to have a significant adverse impact on health outcomes [7]. Although interpreter systems in hospitals have improved over the years, the necessity of an interpreter means that communication is intrinsically compromised in these encounters. Specifically, interpreters can have an inaccurate assessment of a patient's affect and thought process, which can cause misunderstandings between patients and providers [8]. Communication with our patient involved the challenges of interpretation from English to Haitian-Creole, and the COVID-19 precautions required primarily virtual interface, adding another dimension of difficulty.

Isolation, especially while facing a difficult prognosis, only amplifies the pain a patient feels and can put significant strain on their relationships outside of the hospital. Studies found that patients who experience more challenges in interpersonal communication reported lower physical and mental health-related quality of life [9]. The patient was isolated while undergoing very difficult counseling about her high-risk pregnancy and coping with the news that her fetus had a minimal chance of survival. An inability to connect with family and support persons influenced her appetite, mood, and energy level.

Obstetrically, barriers to pregnancy care became glaringly evident during our patient's admission. Negative pressure isolation rooms require appropriate PPE precautions and standards to be performed before entering the room. Naturally, limiting the number of times a nurse or provider enters the room helps limit potential provider exposure as well as conserve resources in a time of high demand and decreased supply. However, the downstream effect of limiting in-person contact with healthcare providers can result in a worsened quality of care. Subtle clinical changes in the obstetric patient, including changes in color and odor of vaginal discharge, mild uterine tenderness (both signs of uterine infection), and even decreased fetal movement, may easily be overlooked.

From a nursing perspective, subtle clinical changes in a patient's care are more likely to be evaluated efficiently when a nurse has developed a rapport and an understanding of the patient's clinical baseline by being at their bedside. Clinical evidence has demonstrated that effective patient-provider communication allows for a trusting relationship that reduces the risk of adverse events [9]. Without regular contact with patients, providers are less likely to pick up on these subtle signs, leading to a delay in response to obstetric emergencies.

Delays in obstetrical response can have a profound effect on maternal and neonatal outcomes. For example, if the patient is undergoing daily fetal monitoring and is found to have fetal bradycardia, this would require immediate resuscitative efforts, including fluid bolus, oxygen supplementation, and left lateral tilt maternal positioning. If the bradycardia persists with no improvement, emergent cesarean delivery may be warranted. Ordinarily, the nurses and physicians on the unit would enter the room and act immediately. During this pandemic, however, all providers would first be required to remove all personal items (including phones and pagers) and then don appropriate PPE, a process which can take 5-10 minutes. Once in the patient's room, communication tools are severely limited. Video conferencing would be required to communicate with labor and delivery staff and anesthesia, neonatology, and operating room staff. Essentially, mobilizing the patient quickly to the operating room would require a much more coordinated effort to ensure safe and quick transport.

In conclusion, obstetric healthcare providers may not be able to provide the highest quality care during the COVID-19 pandemic with the essential precautions in place. Further investigation is required to determine how we can prevent transmission without sacrificing quality patient care. As more information is gathered on SARS-CoV-2, we are hopeful that patients will only be isolated if absolutely necessary to prevent the spread of disease, rather than out of an abundance of caution.

## References

[1] Cascella M., Rajnik M., Cuomo A., Dulebohn S. C., Di Napoli R. (2020). *Features, evaluation and treatment coronavirus (COVID-19)*.

[2] Pregnant I. Y. A. Breastfeeding, or caring for young children | CDC. https://www.cdc.gov/coronavirus/2019-ncov/need-extra-precautions/pregnancy-breastfeeding.html.

[3] Wilson C. C. (2013). Patient safety and healthcare quality: the case for language access. *International Journal of Health Policy and Management*.

[4] Hurtig R. R., Alper R. M., Berkowitz B. (2018). The cost of not addressing the communication barriers faced by hospitalized patients. *Perspectives of the ASHA Special Interest Groups*.

[5] Krishnasamy R. (2019). Contact precautions for colonisation with multidrug-resistant organisms and haemodialysis patient quality of life and mood: a pilot case-control study. *Renal Society of Australasia Journal on*.

[6] Benza S., Liamputtong P. (2014). Pregnancy, childbirth and motherhood: a meta-synthesis of the lived experiences of immigrant women. *Midwifery*.

[7] Flores G. (2016). The impact of medical interpreter services on the quality of health care: a systematic review. *Medical Care Research and Review*.

[8] Diamond L., Izquierdo K., Canfield D., Matsoukas K., Gany F. (2019). A systematic review of the impact of patient–physician non-English language concordance on quality of care and outcomes. *Journal of General Internal Medicine*.

[9] Samuel C. A., Mbah O., Schaal J. (2020). The role of patient-physician relationship on health-related quality of life and pain in cancer patients. *Supportive Care in Cancer*.

